# Nutrient Removal and Recovery from Urine Using Bio-Mineral Formation Processes

**DOI:** 10.1021/acssusresmgt.4c00025

**Published:** 2024-09-13

**Authors:** Robert E. Colston, Ajay Nair, Peter Vale, Francis Hassard, Tom Stephenson, Ana Soares

**Affiliations:** † Cranfield Water Science Institute, Cranfield University, College Road, Cranfield MK43 0AL, UK; ‡ Microvi Biotech, 26229 Eden Landing Rd, Hayward, California 94545, United States; § Severn Trent Plc. Severn Trent Centre, 2 St John’s Street, Coventry CV1 2LZ, UK

**Keywords:** nutrient recovery, urine treatment, bio-based economy, bio-mineralization, struvite

## Abstract

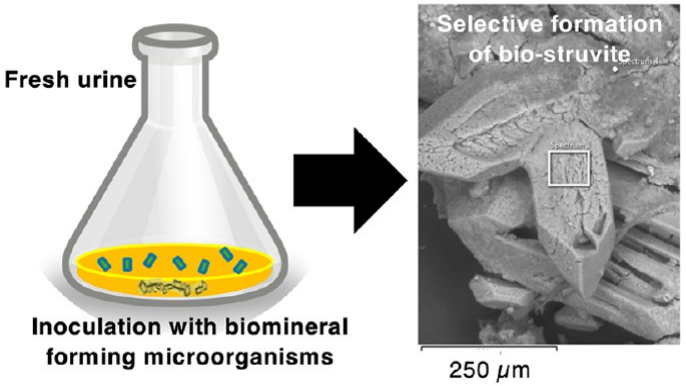

Harvesting nutrients from waste presents a promising initiative to advance and deliver the circular economy in the water sector while mitigating local shortages of mineral fertilizers worldwide. Urine, a small fraction of municipal wastewater, holds substantial amounts of nitrogen, orthophosphate (PO_4_–P), and chemical oxygen demand (COD). Separating urine aids targeted nutrient recovery, emissions reduction, and releasing capacity in wastewater treatment plants and taps into overlooked vital nutrients like magnesium (Mg^2+^) and potassium (K^+^), essential for plant growth. The ability of selected microorganisms (*Brevibacterium antiquum, Bacillus pumilus, Halobacterium salinarum, Idiomarina loihiensis*, and *Myxococcus xanthus*) to remove and recover nutrients from fresh urine through bio-mineral formation of struvite was investigated. The selected microorganisms outcompeted native microbes in open-culture fresh urine, and intact cell counts were 1.3 to 2.3 times larger than in noninoculated controls. PO_4_–P removal reached 50% after 4 days of incubation and 96% when urine was supplemented with Mg^2+^. Additionally, soluble COD was reduced by 60%; urea hydrolysis was only < 3% in controls, but it reached 35% in inoculated urine after 10 days. The dominant morphology of recovered precipitates was euhedral and prismatic, identified using energy dispersive spectroscopy and X-ray diffraction as struvite (i.e., bio-struvite), but K^+^ was also present at 5%. Up to 1 g bio-struvite/L urine was recovered. These results demonstrate the ability of bio-mineral producing microorganisms to successfully grow in urine and recover nutrients such as bio-struvite, that could potentially be used as sustainable fertilizers or chemicals.

## Abstract 2

Urine, rich in N and P water pollutants, served as a substrate for microorganisms to recover nutrients via bio-mineral formation recovering resources sustainably.

## Introduction

Improving the sustainability of food production through nutrient recovery from urine^1,2^ is a growing area of research. Urine only contributes to 1% of municipal wastewater, yet it contributes to 80% of nitrogen (N), 50% of orthophosphate (PO_4_–P), and 10% of chemical oxygen demand (COD).^3,4^ As such, treating urine separately has been inferred to increase the efficiency of nutrient recovery and reduce overall greenhouse gas emissions and costs to wastewater treatment plants (WWTPs).^2,5^ Additionally, urine is rich in other key nutrients required for plant growth, i.e., magnesium (Mg^2+^) and potassium (K^+^), but usually these are overlooked. Life-cycle assessment has proven that source separating urine and treatment can reduce the global warming potential, eutrophication potential, and cumulative energy demand up to 63% by reducing freshwater use and nutrient-load to WWTPs.^1,2^ This reduces the overall energy required for wastewater treatment, and recovered fertilizers can offset greenhouse gas emissions from the production and transport of synthetic/mined fertilizers.^2^ There are many novel processes to treat urine (Table 1) with many relying on the first step of volume reduction using renewable dehydration media (i.e. wood ash)^6^ or, alternatively, through reverse osmosis (Table 1), which can reach a flow of 11.9 ± 1 L/day·m^2^ with >70% N and 100% phosphorus (P) and K^+^ recovered. The retentate of some technologies, such as volume reduction and ion exchange processes, can be used as a liquid fertilizer^6^ (Table 1). In microbial fuel cells, electrons and protons released from the microbial oxidation of organic matter within the urine are transferred between an anode and cathode to generate electricity, which can be used to power selective electrodialysis to remove nutrients such as PO_4_–P^4,7^ (Table 1). In developing countries where dry toilets have been installed as a decentralized option for safe sanitation, struvite recovery from urine could provide a source of renewable fertilizer for local use or resale.^8^ Struvite has been proven to be a slow-release fertilizer, increasing research into recovering struvite as a sustainable fertilizer alternative.^9^ Issues remain due to the value of chemical struvite not offsetting the cost of its recovery in traditional precipitation reactors to overcome the cost of mined mineral fertilizers. Further to this, urine can contain micropollutants and pharmaceuticals,^10^ further decreasing the value of recovered products. The processes described in Table 1 have reached different stages of development and implementation; however, the costs of materials and energy demand remain high for most.^7,19,20^

**Table 1:** Urine Treatment Processes Producing Fertilizer Alternatives

**Process**	**Method**	**Advantages**	**Disadvantages**	**Ref**
**Nutrient precipitation-Magnesium based**	Addition of MgO to increase saturation of struvite, most efficient when the Mg:P ratio is raised to 2:1 combined with a pH control	99% PO_4_–P recovery at room temperature and chemical struvite fertilizer alternative	3–5% N-recovery pH control is required to maintain a value of 9	(1,11,12)
**Nutrient precipitation-Calcium based**	Ca(OH) was used to simultaneously increase the pH and increase the saturation of Ca to encourage precipitation of hydroxyapatite (HAP)	Up to 95% PO_4_–P recovery as Ca_3_(PO_4_)_2_ or HAP at 25 °C, pH 11-13, More stable than struvite in temperatures > 60°C	Hydrolyzed urine promotes CaCO_3_ over HAP due to abundance of CO_3_^2–^ from complete urea hydrolysis	(1,13,14)
**Nutrient concentration-Microbial fuel cells + selective electrodialysis**	Microbial oxidation in urine generates electricity to power nutrient concentration via electrodialysis	Allows for removal of 97.4% NH_4_–N and multiple ions from pre-treated urine (76.7% PO_4_^3–^, 94.5% SO_4_^2–^ )	A pretreatment step is needed and scale-up is required with optimized nutrient recovery	(4,7)
**Nitrogen recovery-Ammonia stripping/scrubbing**	Induced volatilization of NH_4_^+^ through the pH and temperature change. Bubble NH_3(g)_ through weak acid (sulfuric or acetic acids), adsorbing NH_3_	NH_4_^+^ recovery up to 95%, final product depends on the acid used	Possible NH_4_^+^ losses during storage or transport of the urine; Difficulty removing ammonia from absorption media; If ion exchange is used, regeneration can be complex	(1,15,16)
**Nitrogen recovery-Adsorption**	High electronegative materials are used to sorb NH_4_^+^, Materials used: zeolites, hybrid ion exchange resins (HIXR) (Fe-based), or activated carbon (coconut shell, sawdust, charcoal)	Besides N recovery, up to 92% P recovered using HIXR, Removed 256 mg N/g coconut shell = 95% ammonia recovery		
**Volume reduction-Evaporation**	Heat source (solar, burning biogas) evaporates the water fraction, increasing the concentration of nutrients in the remaining liquid. The condensate can be used as water for reuse.	Up to 95% water recovery and 95% NH_4_^+^ recovery	NH_4_^+^ volatilization increases, up to 93% loss; High energy demand; Risk of concentrating pharmaceuticals, parasites, pathogens, and heavy metals in liquid fertilizer products	(1,6,17,18)
**Volume reduction-Reverse osmosis**	Overpressure influent, increasing hydrostatic pressure drawing water from an influent through a membrane into a buffer solution	Potable water (80% volume reduction), Nutrient-rich supernatant can be used as liquid fertilizer, 1/2 the cost of evaporation techniques	Filter clogging; Risk of concentrating pharmaceuticals, parasites, pathogens, and heavy metals in liquid fertilizer products	
**Volume reduction-Freeze concentration**	Effluents chilled to −30 °C to reach the eutectic point of salts to cause precipitation, Suggested to use after preconcentration effluents using reverse osmosis and before struvite recovery	99% salts recovered and 95% water recovery	High energy demand to chill wastewater to −30 °C; Risk of concentrating pharmaceuticals, parasites, pathogens, and heavy metals in liquid fertilizer products	

Furthermore, chemical recovery processes typically involve a two-stage treatment approach, entailing stabilizing or hydrolyzing urea in urine, whilst minimizing ammonia volatilization, thereby amplifying the demand for urine storage at treatment sites, followed by the chemical-driven recovery process.^1,14,21^ Urea hydrolysis (ureolysis) or stabilization is required to ensure consistent influent quality, transport urine, and improve the ease of the controlling pH as ureolysis causes the pH to increase leading to ammonia volatilization.^22^ Stabilization of urea requires acidification or alkalinization to bring the pH of the urine to below 4 or above 11, to denature any free urease enzymes and prevent hydrolysis of urea.^6,14,19,23^ Without the presence of enzymes, urease producing bacteria (abound in urine collection systems, fostering biofilm formation on pipes), and initiating urea hydrolysis, or chemical addition, complete ureolysis is a lengthy process, with an estimated half-life of 1.5-3.6 years at 25 °C, depending on the concentration of urea.^14,19,21,24^ To accelerate ureolysis, chemicals, bacteria, or enzymes are added, with temperature and/or pH control.^19,21−24^ With the addition of urease enzymes and temperature increase to >50°C, complete ureolysis can be achieved in hours.^14,19^ After stabilization or ureolysis, chemical recovery can take place, which requires the addition of reagents to control the pH, supersaturation of desired ions, and crystal growth. These multiple steps, costs of materials, and energy requirements of such technologies (Table 1) have meant commercial uptake is still slow to reach its full potential. Alternative treatment methods should be investigated that can improve their viability to encourage wider uptake, by providing evidence for effective one-step nutrient recovery that has low energy and reagent requirements, while effectively recovering nutrients. A full review of the technologies and developments on urine treatment and ammonia recovery can be found in the recent publication from Larsen et al., 2021.^25^

The growth of selected microorganisms in wastewater sludge dewatering liquors (SDLs) has been shown to promote the recovery of struvite (henceforth referred to as bio-struvite) through bio-mineral formation without the addition of reagents.^11,26−29^ Bio-mineral formation is the process in which minerals precipitate due to changes in solution chemistry controlled and induced by living organisms. The mechanisms can be split into biologically controlled mineralization (BCM) e.g., magnetosome formation, providing clear benefits to the organism or biologically induced mineralization (BIM) e.g., iron-reducing microorganism causing iron precipitation, which is a byproduct due to chemical changes caused by their metabolic activity. Urine has been inferred to be a potential waste for phosphate (P) and other nutrients’ recovery such as bio-struvite^11,26,27,29^ but not yet investigated in detail. Characterization of five microbial strains known for producing bio-struvite and the mechanisms of precipitation involved was carried out by Simoes et al. and Leng et al. in wastewater.^26,28−32^ Key characteristics of urine are an abundance of carbon sources including urea, which 4 of the 5 microorganisms can utilize as they produce urease,^29^ and COD measures between 5000 and 12000 mg/L in fresh and hydrolyzed urine^33,34^ consisting of other carbon sources such as creatinine, hippuric acid, and citric acid.^29,35^ A public misconception is that urine is sterile, but microbial analysis has shown it is not the case.^36,37^ Despite this, it is perceived that the selected microbes will be able to grow successfully in fresh (untreated), open culture urine,^29^ where open culture refers to native microorganisms present. Due to the availability of PO_4_–P, NH_4_–N, and Mg being greater in urine compared to SDL, the likelihood of bio-struvite recovery taking place would be expected.^31^

This study aims to provide evidence that bio-mineral formation can be applied for nutrient recovery in urine. The five microorganisms have never been studied in fresh urine, and it is not known how native microbes will impact their growth rate, activity, and nutrient removal and what type of bio-mineral would be forming. Each microorganism was inoculated into fresh, untreated urine, and the samples were incubated to monitor their growth. Sacrificial bottles were analyzed throughout the incubation period to analyze changes in urine chemistry and collect precipitates for analysis. The wider implications for this study are providing new data to understand if bio-mineral formation technologies can be applied to treat wastes and recover nutrients as minerals for reuse as fertilizers.

## Materials and Methodology

### Source of Microorganisms and Urine

Microorganisms previously tested and known for their ability to produce bio-struvite in wastewater^29,31^ were selected for this study: *B. antiquum* and *H. salinarum* (DSM 21545 and DSM 671, respectively, German Resource Centre for Biological Material, Germany), *B. pumilus* (GB43, LGC Standards, Middlesex, UK), and *I. loihiensis* and *M. xanthus* (CECT 5996/MAH1 and CECT 422, Spanish Type Culture Collection, University of Valencia, Paterna, Spain).

Fresh urine was sourced through volunteer donations collected in 500 mL pots from both male and female toilets at Cranfield University, typically between 9:00 and 12:00, adhering to established ethical research integrity protocols. Fresh (nonstabilized) urine batches were utilized to grow the selected microorganisms straight after collection or after storage at 4 °C for a maximum of 2 days to limit urea hydrolysis.

### Microbial Incubation in Urine

**Figure 1:**
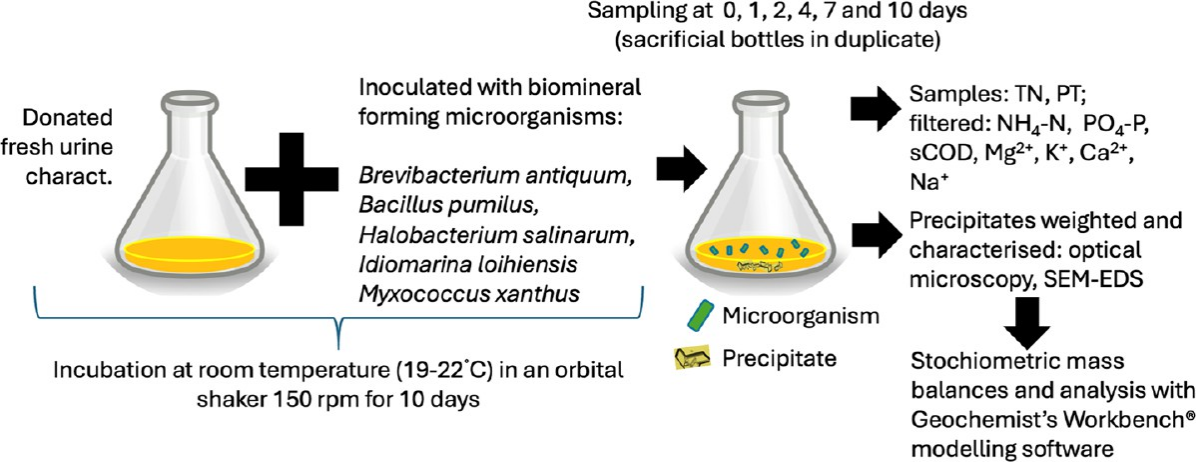
Schematic representation of the overall experimental procedure. In a parallel experiment, *B. antiquum* was also cultivated in fresh urine with magnesium dosing.

Frozen starter cultures of each microbe were inoculated in 300 mL of B4.1 synthetic media (4 g/L of yeast extract, 2 g/L of magnesium sulfate heptahydrate, and 2 g/L of potassium phosphate), incubated in conical flasks at room temperature (19–22 °C), and agitated at 150 rpm (Stuart SSL, Fisher Scientific, Loughborough, UK) under sterile conditions, for 2–3 days to reach the stable growth phase.^29^ After this period, the growth medium was filtered to remove precipitates greater than 10 μm (Whatman, Grade 1 filter sheets), and the microbial cells were centrifuged (Sanyo MSE Falcon 6/300 centrifuge, 2400g, 5 min) from starter cultures and resuspended in the same volume of urine, to avoid the addition of PO_4_, NH_4_–N, and Mg^2+^ present in the B41 media to the urine.^27^ Experiments were completed in batches of sacrificial bottles (i.e., the whole bottle content was used for each sampling point to ensure all precipitates were recovered and analyzed) with 300 mL of urine, inoculated with 50 mL of the filtered and centrifuged starter culture under sterile conditions to ensure only the inoculum introduced the targeted microbes to the raw urine, at room temperature (19–22 °C), and agitated at 150 rpm (Stuart SSL, Fisher Scientific, Loughborough, UK) for 10 days, to ensure bio-minerals are at a recoverable particle size.^26,29^ All experiments were completed in duplicate, in bottles capped with cotton wool stoppers ensuring good aeration, and control bottles contained only urine (noninoculated). A schematic representation of the experiment can be found in Figure 1.

Magnesium sulfate heptahydrate (MgSO_4_·7H_2_O) was added to two additional sacrificial bottles inoculated with *B. antiquum* to observe the impact on PO_4_–P removal and precipitate recovery when Mg was not a limiting nutrient. Based on urine characterization, 15 mL of 0.8 M MgSO_4_·7H_2_O was added to reach a 1.2:1 Mg:P ratio (based on the initial PO_4_–P concentration of the urine batch).

### Analytical Methods

Prior to each incubation, the urine was characterized, and sampling occurred at times of 0, 1, 2, 4, 7 and 10 days of incubation through sacrificial bottles. The pH was measured using a Fisherbrand hydrous 300 pH meter (Fisher Scientific, Loughborough, UK).

The urea content of each batch was estimated based on TN and NH_4_–N measurements using eq 1a, though it is known that this method slightly overestimates due to the presence of proteins, constituting a fraction of organic nitrogen distinct from urea. In all urine batches, nitrate and nitrite levels were below the detection limit, leading to the assumption that nitrates and nitrites were absent, with only ammonia representing the sole form of inorganic nitrogen.^33,34^
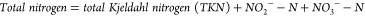
1a
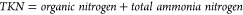
1b
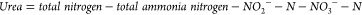
1c

BD Accuri C6 flow cytometry with 488 nm solid-state laser (Becton Dickinson U.K. Ltd., Oxford, UK) was used to measure total cell counts (TCCs), intact cell counts (ICCs), and proportion of high nucleic acid (HNA) to low nucleic acid (LNA) in the urine at the same sampling intervals using SYBR Green I and propidium iodide staining.^38^ Instrument noise was accounted for according to Gatza et al. (2013) using BD Accuri C6® software.^39^

Stochiometric mass balances and the Geochemist’s Workbench® modeling software were used to pinpoint abiotic mineral precipitates during urine incubation, using thermodynamic modeling (PHREEQC, US Geological Society (USGS)) based on the activities of measured ions and the environmental conditions (pH, temperature, and pressure = 1 bar). The measured ions (Mg^2+^, Ca^2+^, and K^+^) provided estimates for struvite, calcium phosphate, potassium phosphate, and the total precipitates. The mass balances considered changes in these measured ions at a stable pH, which would support abiotic nucleation in controls.

Precipitates were recovered from sacrificial bottles using vacuum filtration through a 10 μm filter paper (Whatman, Grade 1 filter sheets), dried at room temperature (19–22 °C), and weighed. The quality of precipitates, their assemblage, and morphological characteristics were analyzed using optical microscopy (Olympus MX40) and scanning electron microscopy (SEM) coupled with energy dispersive X-ray spectroscopy (EDS) to distinguish the mineralogy using point ID analysis and element mapping (Tescan Vega 3, Oxford Instruments© AZtecCrystal, Abingdon, United Kingdom). Powdered X-ray diffraction (XRD) was used to support SEM-EDS analysis by comparing precipitate spectra with pure struvite and calcium phosphate spectra (Siemens D5005, Manchester, United Kingdom).

## Results

### Initial Urine Characterization

The characterization of each urine batch (UB) is presented in Table 2. On average, the pH was slightly acidic ranging from 5.76 to 6.42. There was high variability in the concentrations of sCOD, NH_4_–N, and TN among the batches collected (Table 2); however, the ratio between sCOD and TN remained stable at 2 in UB1 and UB5 (representing the weakest and strongest loaded batches collected), and PO_4_–P was affected also by a factor of 2 from UB1 to UB5. Soluble chemical oxygen demand varied from 5740–15140 mg/L, NH_4_–N varied from 152–595 mg/L, and TN varied from 820–3250 mg/L (Table 2). PO_4_–P and TP were very similar in all batches, PO_4_–P varied from 228–466 mg/L, and TP varied from 270–515 mg/L (Table 2). Magnesium, Ca^2+^, and K^+^ were relatively consistent across all urine batches recording values of 32–63 mg Mg^2+^/L, 66–111 mg Ca^2+^/L, and 1048–1992 mg K^+^/L (Table 2).

**Table 2:** Chemical Characterization of Different Urine Batches (UBs) Collected and Measured in Duplicate (Average ± Standard Deviation) and the Corresponding Microbe Inoculated

	**pH**	**SCODmg/L**	**NH**_**4**_**–Nmg/L**	**TNmg/L**	**PO**_**4**_**–Pmg/L**	**TPmg/L**	**Mg**^**2+**^**mg/L**	**Ca**^**2+**^**mg/L**	**K**^**+**^**mg/L**	**Inoculated microorganism**
**UB1**	6.42 ± 0.00	5740 ± 100	152 ± 8	3250 ± 50	228 ± 1	270 ± 14	32 ± 2	66 ± 1	1048 ± 2	*B. antiquum*
**UB2**	6.09 ± 0.00	7140^a^	316^a^	3500^a^	424^a^	384^a^	48 ± 4	92 ± 0	1772 ± 2	*B. pumilus*
**UB3**	5.76 ± 0.01	9290 ± 30	595 ± 11	4940 ± 60	386 ± 12	386 ± 8	56 ± 1	111 ± 5	1778 ± 21	*H. salinarum*
**UB4**	5.86 ± 0.00	8200 ± 320	404 ± 12	7650 ± 150	420 ± 20	515 ± 15	63 ± 1	100 ± 24	1394 ± 20	*I. loihiensis*
**UB5**	6.11 ± 0.00	15140 ± 380	390 ± 35	8250^a^	466 ± 6	467 ± 5	62 ± 1	73 ± 1	1992 ± 30	*M. xanthus*

a Single measurement.

### Microbial Growth in Urine

**Figure 2:**
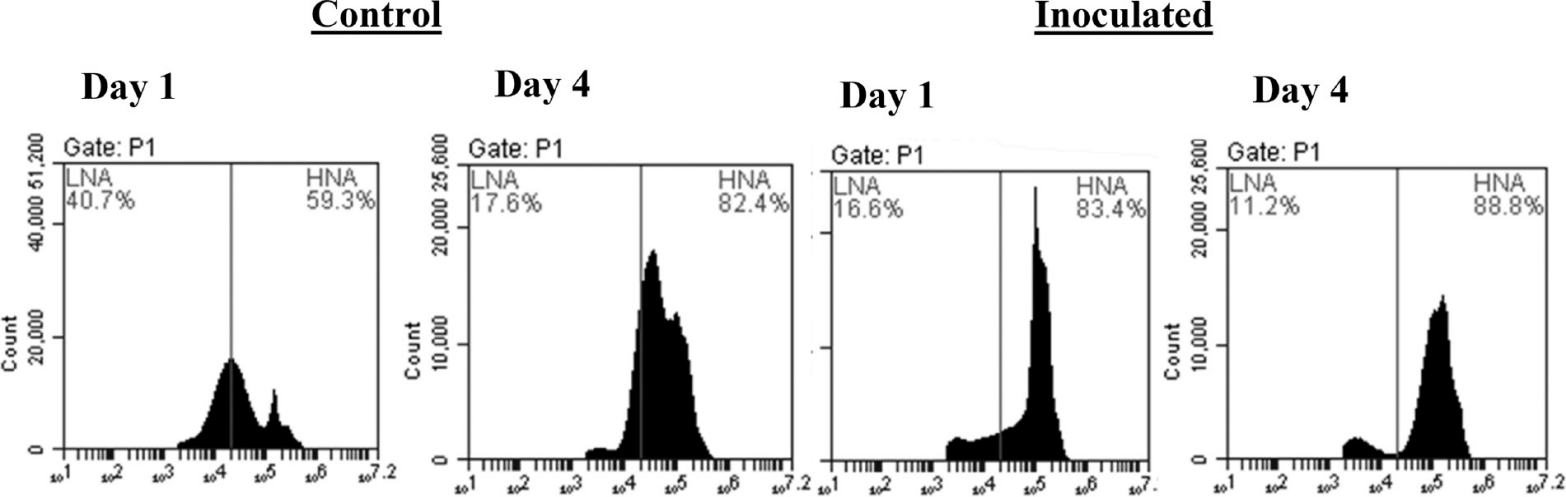
Typical change in proportion of HNA to LNA between controls (left) and inoculated urine batches (right, data for *B. pumilus*) after 1 and 4 days of incubation.

**Figure 3:**
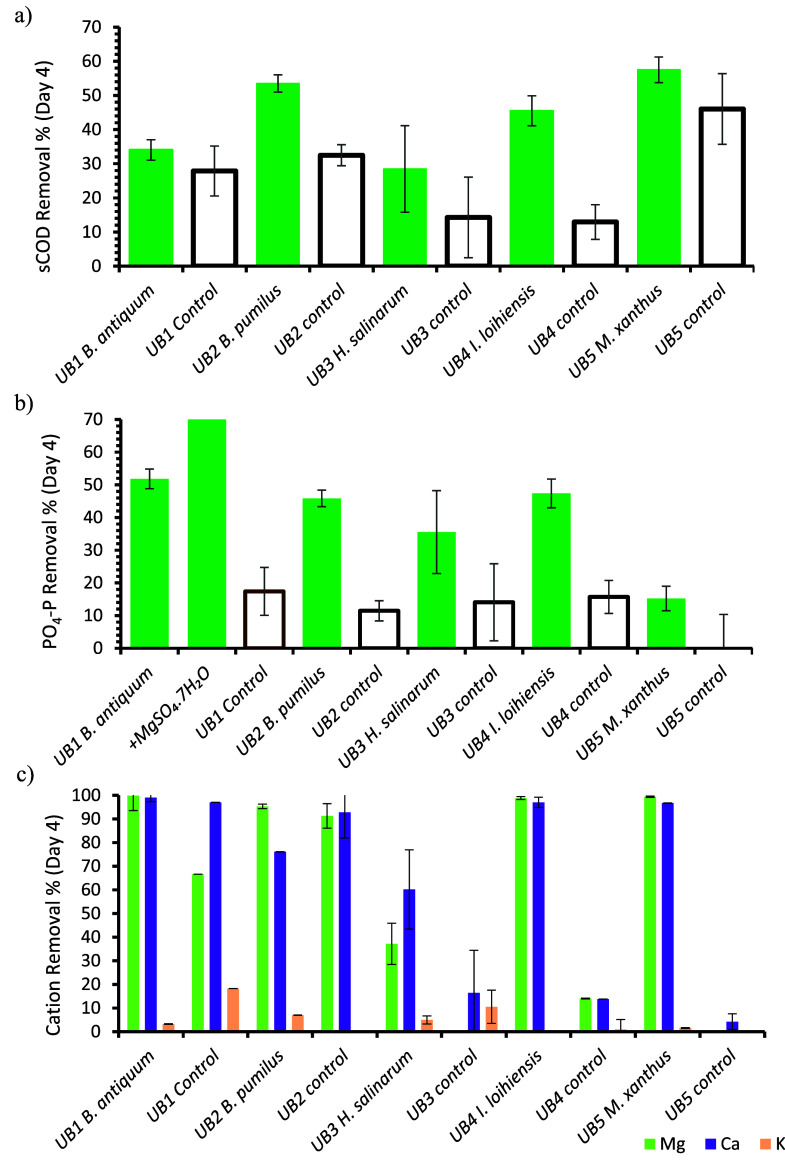
Removal percentages from the starting concentration (Table 2) by day 4. a) sCOD, b) PO_4_–P, including removal when dosed with magnesium sulfate to *B. antiquum*, and c) major cation (Mg, Ca, K) removal.

The microbial growth within inoculated and control urine bottles was observed by measuring intact cell counts (ICCs) shortly after inoculation (Figure Si-1). Initial ICCs in urine varied from 4.25 × 10^6^ to 3.64 × 10^10^ ICC/mL, which increased from 6.17 × 10^8^ to 3.16 × 10^12^ in the controls and 7.36 × 10^8^ to 5.56 × 10^12^ ICC/mL in inoculated bottles after the first day of incubation (Figure Si-1). Tests showed an increase in the ICC in the inoculated bottles, 1.3 to 2.73-fold higher than in the controls (Figure Si-1). After the first day of incubation, the ICC in the inoculated tests and controls stabilized (Figure Si-1). The specific growth rates of the microorganisms in inoculated tests were between 0.09–0.18 L/h and 0.07–0.14 L/h in the controls, during the first day of incubation. From day 2 onwards, the ICC remained constant, and the growth rate was close to zero. High nucleic acid versus LNA percentages highlighted key differences between the controls and inoculated tests (Figure 2). In the controls, percentages of HNA increased throughout the incubation period starting between 45% and 60% at day 1, increasing up to 85% by day 4. Inoculated tests showed a larger proportion of HNA compared to controls by day 1, measuring between 80% and 90% HNA (Figure 3). In the inoculated bottles, HNA stayed between 80–90% throughout the incubation period.

### Solution Chemistry

The pH in the inoculated tests and controls increased throughout the incubation period, rising to between 7.1 and 9.5 (Table Si-1). In inoculated tests, sCOD was reduced by 28–57% (1953 to 3820 mg/L) (Figure 3a), whilst in controls, this was 13–46% (1060 to 3275 mg/L) by day 4 (Figure 3a) (Figure Si-2). By day 10, sCOD removal in the controls was 39–66%, while in inoculated tests, removal reached 69%. Orthophosphate removal ranged between 15–52% (71 to 199 mg/L) for inoculated tests by day 4 (Figure 3b), narrowing to 28–52% by day 10. *Myxococus xanthus* measured the poorest PO_4_–P removal of 15% by day 4, whilst *B. antiquum* achieved 52% removal in 4 days of incubation (Figure 3b). In the controls, the PO_4_–P removal percentages were much lower, between 0–17% (0 to 66 mg/L) by day 4 (Figure 3b), increasing to between 11 and 34% by day 10.

When urine was inoculated with *B. antiquum* and supplemented with Mg^2+^, a PO_4_–P removal of 96% (219 mg/L) was measured, compared to 52% without Mg^2+^ (Figure 3b) (Figure Si-2). Soluble chemical oxygen demand removal increased to 49% compared to 39% in *B. antiquum* bottles without Mg^2+^ added. Values of the pH, TN, TP, and NH_4_–N had little to no difference compared to those of inoculated tests without supplemented Mg^2+^. The Mg^2+^ and Ca^2+^ removals in inoculated tests were approaching 100%, and by day 4, close to all Mg^2+^ was removed with most also showing near complete Ca^2+^ removal (Figure 3c). Potassium removal reached 5% in *B. antiquum, B. pumilus*, and *H. salinarum* (Figure 3c). In controls, cation removal was much more varied; in UB1 and UB2, Ca^2+^ removal was close to 100%, whereas in UB3, UB4, and UB5, it was <20%. In all controls, Ca^2+^ removal was higher than Mg^2+^ and K^+^, apart from UB2 whose Mg^2+^ removal was similar to Ca^2+^ (Figure 3c).

**Figure 4:**
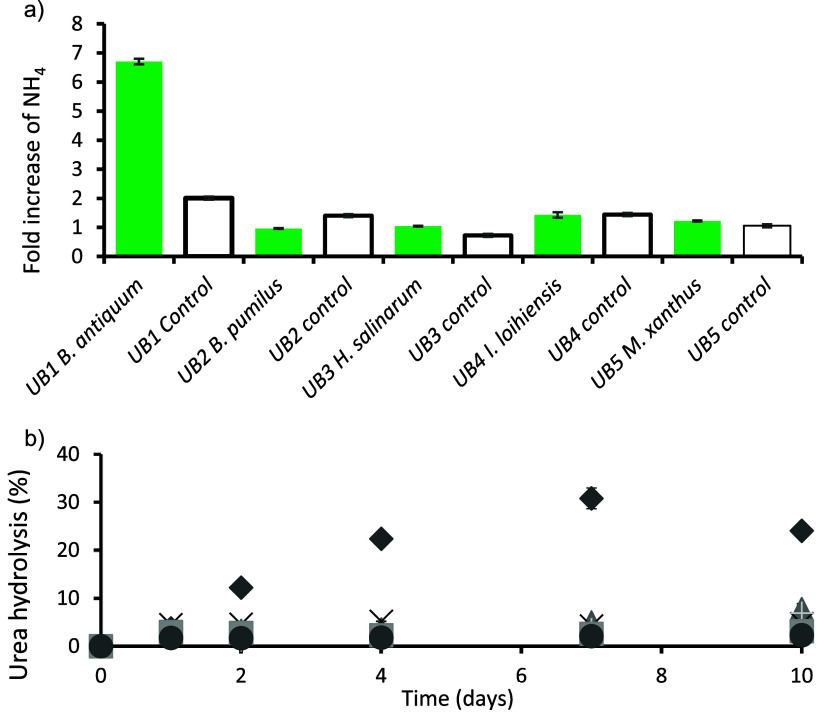
a) Fold increase of ammonia by day 4 and b) hydrolyzed urea during incubation. UB average for all controls (○), UB1 *B. antiquum* (◆), UB2 *B. pumilus* (▲), UB3 *H. salinarum* (×), UB4 *I. loihiensis* (+), and UB5 *M. xanthus* (■).

Ammonia increased throughout the incubation period across all urine batches (Figure 4a) (Figure Si-2). In *B. antiquum*, NH_4_–N increased by 6.5-fold (from 152 mg/L to 1870 mg/L, by day 4) and by 16-fold by day 10. In the other inoculated tests, NH_4_–N increased by 3-fold, whilst in the controls, it was up to 2-fold by day 4 (Figure 4a). Nitrate and nitrite were always below the detection limit, when measured at various points during the incubation period, indicating that nitrification did not occur.

The initial urea concentration in the various urine batches was estimated to be between 14.6–39.4 g N/L urine. Based on these estimates, the percentage of urea hydrolyzed was calculated (Figure 4b). All microorganisms tested showed the ability to hydrolyze urea when compared to their respective controls, except for *I. loihiensis*. The bottles inoculated with *B. antiquum* recorded the highest urea hydrolysis, reaching 32% by day 7 (Figure 4b).

### Mass Balances

**Figure 5:**
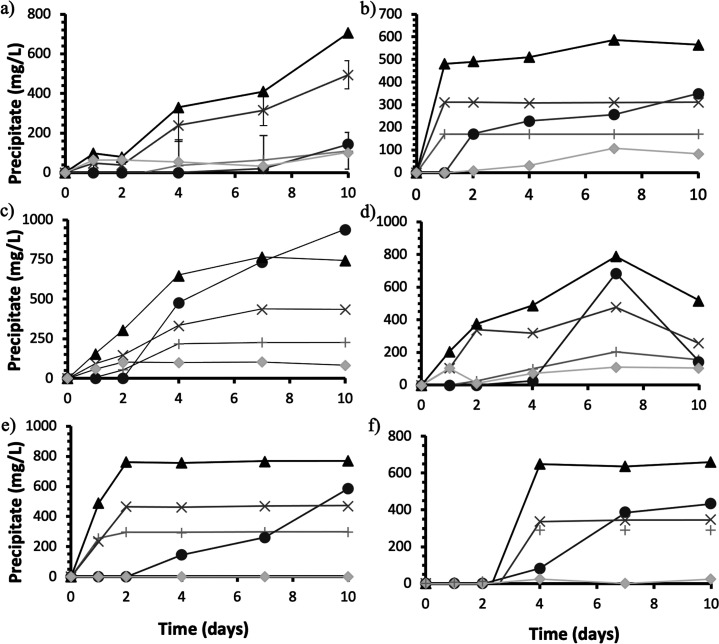
Estimated mineral assemblages collected from UB. Struvite (+), calcium phosphate (×), potassium phosphate (◆), sum of precipitate estimates (▲), and actual precipitate measured (●): a) average UB control, b) *B. antiquum*, c) *B. pumilus*, d) *H. salinarum*, e) *I. loihiensis*, f) *M. xanthus*.

Stochiometric mass balances of the measured ions throughout the incubation period, in conjunction with geochemical model software, were used to identify abiotic mineral precipitates during urine incubation (Figure 5). The assemblages considered in this study included struvite (Mg(NH_4_)PO_4_·6H_2_O), calcium phosphate (Ca_3_(PO_4_)_2_), and potassium phosphate (K_3_PO_4_) plus the total precipitate estimate and the actual mass of precipitates recovered. The estimated total precipitates’ mass in the controls was 706 mg/L urine by day 10, whilst the actual mass recovered never exceeded 103 mg/L urine. The precipitates were recovered after filtration through a 10 μm membrane, and smaller crystals would not be accounted for; but the difference between measured and estimated seems too high. Calcium phosphate accounted for 70% of the control precipitate by day 10 (Figure 5a). In inoculated tests, the total precipitate calculated also exceeds the actual precipitate recovered, except for *B. pumilus* 7 (Figure 5c). In inoculated tests, struvite was calculated to account for 30–49% of all precipitates and exceeded the average control UB estimate of 15%. The predicted formation of K_3_PO_4_ in *B. antiquum, B. pumilus*, and *H. salinarum* was between 12–20% and 15% in controls (Figure 5b-d).

**Figure 6:**
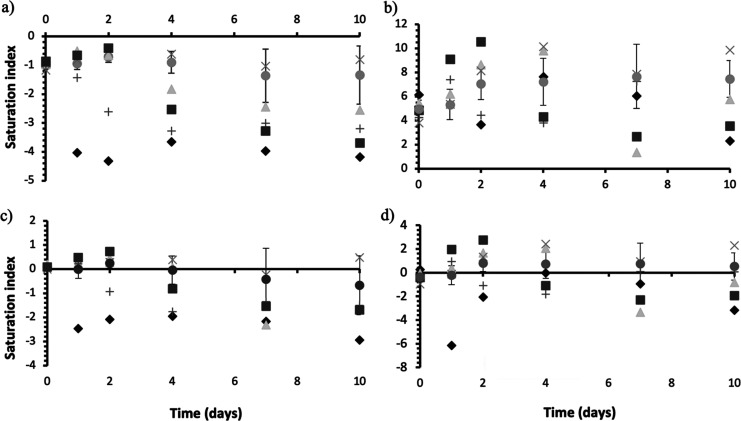
Saturation indices calculated through the Geochemist’s Workbench®. UB average (●), *B. antiquum* (◆), *B. pumilus* (▲), *H. salinarum* (×), *I. loihiensis* (+), *M. xanthus* (■). a) struvite, b) hydroxyapatite, and (c-d) other calcium phosphate minerals.

Using the pH, temperature, and chemical data collected together with the thermodynamic model from the PHREEQC data set (USGS), the saturation indices for precipitation of struvite, hydroxyapatite, and calcium phosphate were calculated over the incubation period (Figure 6). Saturation indices (SIs) are calculated following the laws of thermodynamics,^40^ to predict what minerals can be precipitated from a system following the simple rules in eq 2a. The saturation index of a mineral was calculated from the ionic activity of the ions present and their solubility constants (*K_sp_*) for the environmental conditions of the reaction (temperature, pH, and pressure).

Ionic activity is dependent on the concentration of ions, temperature, pH, and pressure following the Debye-Hückel equation; in environmental chemistry, this is simplified to assume temperature is equal to 298 K and pressure is constant at 1 bar.^40^ Using the Debye-Hückel equation and eq 2a, the SI can be calculated and predicts whether precipitation will occur if the mineral is supersaturated, SI > 0, or if dissolution will occur as the mineral is undersaturated, SI < 0 (eq 2a).
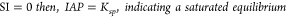
2a
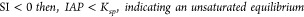
2b
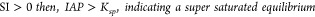
2c

Figure 6 shows that hydroxyapatite and other calcium phosphate minerals have positive saturation indices in all urine bottles (inoculated and controls; Figure 6b-c), indicating they are the most likely minerals to precipitate abiotically from solution based on thermodynamic principals. The saturation indices for struvite remain negative throughout the incubation period in all bottles (Figure 6a), indicating that abiotic struvite was not expected to precipitate.

### Precipitate Recovery and Characterization

Across all inoculated tests, precipitates > 10 μm were collected through filtration and exceeded the recovered precipitate weight recovered from the control bottles. Precipitates in inoculated tests were observed as early as day 2 and were recoverable by day 4. In *B. antiquum* tests, the precipitates could be recovered from day 2 onwards (Figure Si-2). In controls, no precipitates > 10 μm were recovered before day 7. After 10 days of incubation, the weight of recovered precipitates varied between 330–1000 mg precipitate/L urine in the inoculated tests and between 54–168 mg precipitate/L urine in controls. *Bacillus pumilus* tests had the highest recovery of 1000 mg of precipitate/L urine. In bottles with *H. salinarum*, 680 mg of precipitate/L urine was recovered by day 7 but decreased to 142 mg/L urine at day 10. This resolubilization of the precipitates was only observed for this microbe and is supported by an increased Mg^2+^ concentration measured in solution during that time (Figure 5d).

**Figure 7:**
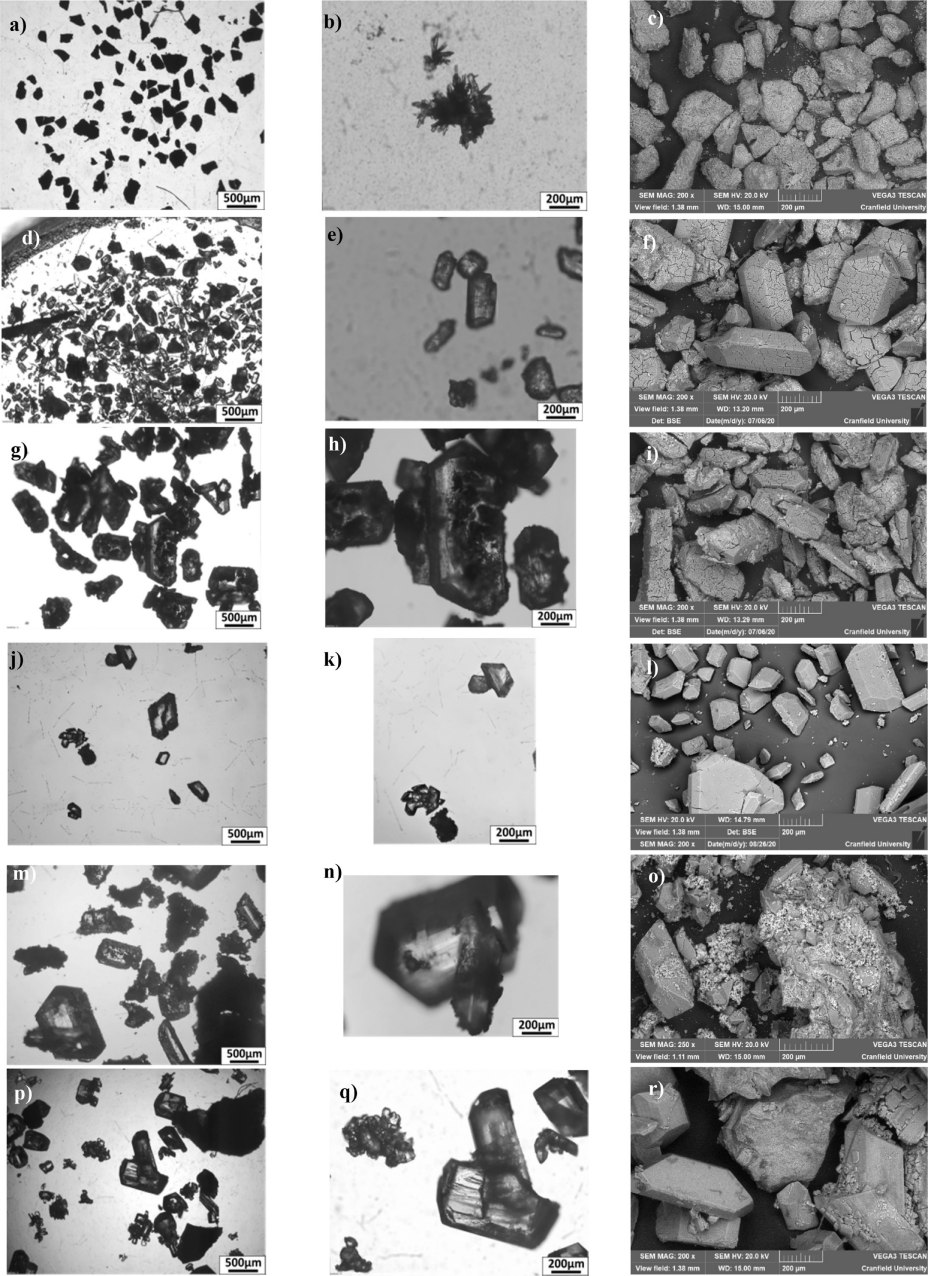
Optical microscopy and SEM images of precipitates collected after 4 days of urine incubation. Images from a) to c) are controls, d) to f) *B. antiquum*, g) to (i) *B. pumilus*, j) to l) *H. salinarum*, m) to o) *I. loihiensis,* and p) to r) *M. xanthus*.

Microscopy and optical microscopy revealed differences between the mineral assemblages recovered from the controls and inoculated tests (Figure 7). Prismatic and tabular crystals that were well formed (euhedral) and translucent were recovered from all microorganism tests (Figure 7d-r). However, the assemblages from *I. loihiensis* and *M. xanthus* had euhedral crystals held within amorphous precipitates (Figures 7o and 6r), whereas *Brevibacterium antiquum*, *B. pumilus*, and *H. salinarum* had less abundant and smaller amorphous crystals than typically coated the larger euhedral crystals. In contrast, the mineral assemblages from controls were dominated by amorphous precipitates, which were opaque under optical microscopy, with minor translucent acicular crystals (Figure 7a-c).

**Figure 8:**
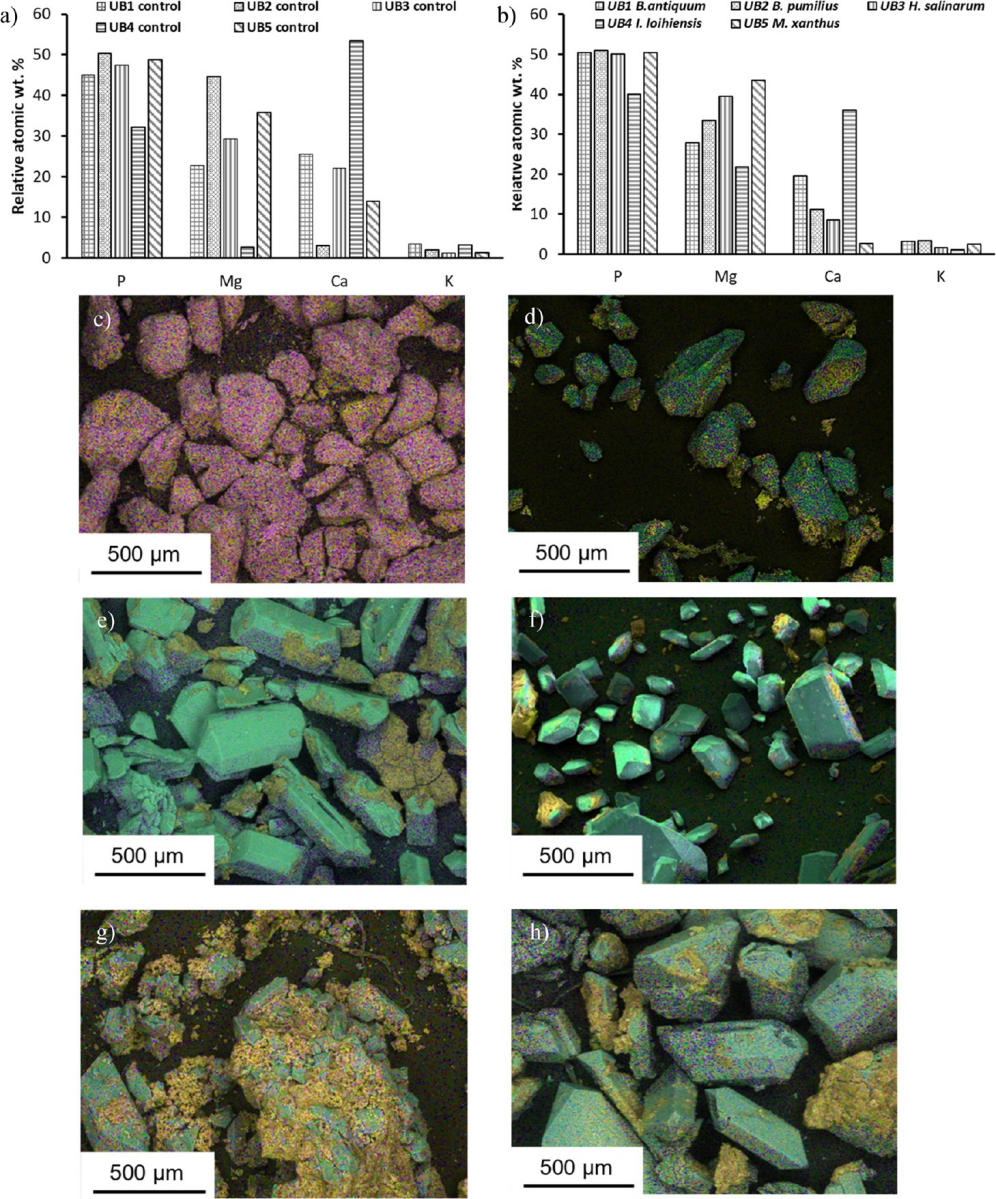
Scanning electron microscopy with EDS of the precipitate assemblages after incubation for 10 days of incubation. Relative atomic weight spectra of a) UB controls and b) UB inoculated with microorganisms. Element mapping of precipitate assemblages where pink is P-rich, yellow is Ca-rich, and green is Mg-rich, for c) control precipitates, d) *B. antiquum*, e) *B. pumilus*, f) *H. salinarum*, g) *I. loihiensis*, h) *M. xanthus*.

Scanning electron microscopy with EDS of the precipitates recovered from inoculated tests measured a higher proportion of Mg than controls, reaching up to 40 wt % and averaging at 36 wt % (Figure 8a-b). Phosphorus accounted for 50 wt % of precipitates recovered from inoculated tests, except for *I. loihiensis* whose P wt % was 40. The measured ratio of P:Mg is close 1:1 and indicated most precipitates from inoculated tests are bio-struvite (MgNH_4_PO_4_·6H_2_O) (Figure 8b).

Element mapping of precipitates showed different element associations across the bimodal distribution of morphologies (Figure 8c-n). Precipitates from inoculated tests showed a clear distinction between amorphous crystals and prismatic crystals, with Ca (in yellow-pink) and Mg (in turquoise) aligned, respectively (Figure 8d-h). The abundance of Ca-minerals within each precipitate assemblage varied, and *B. antiquum, B. pumilus, H, salinarum*, and *M. xanthus* presented some Ca-minerals coating bio-struvite precipitates, whilst *I. loihiensis* precipitates show struvite within amorphous masses of calcium phosphate (Figure 8g). Precipitates collected from controls had a higher proportion of Ca (up to 54 wt %) compared to Mg, which fluctuated between 3 and 45 wt %, averaging at 27 wt % (Figure 8a). Measured P was between 32–50 wt % in control precipitates. Element mapping showed no clear differentiation between Ca assemblages, Mg assemblages, and P in controls (in yellow to pink) (Figure 8c).

## Discussion and Conclusions

The fresh urine investigated in this study showed significant variability in the pH, sCOD, and key cation concentrations in between batches but presented similar ratios of sCOD:TN of 2, and the TP:Mg2+ was also similar to all batches at an average of 7.4 (Table 2). Compared to previous studies also using fresh urine,^3^ the streams tested were weaker in strength with respect to sCOD, PO_4_–Pd, and Mg^2+^ in the study by Rose et al. (2015). By the end of the 10-day incubation, urea hydrolysis in controls was measurable; however, the final characterization showed it was far from completely hydrolyzed urine,^33^ which was to be expected considering the rate of urea hydrolysis measured in other studies at room temperature and with no pH adjustment.^14,24^ Nevertheless, to better estimate hydrolysis rates, it is recommended that urea is measured directly in urine, instead of estimating it by measuring nitrogen rich species such as TN and NH_4_–N (eq 1a).

As the microorganisms investigated were inoculated in different nonsterile fresh urine batches with different concentrations of carbon and nutrients from the start, comparisons between microorganisms need to be done carefully, but a direct comparison can be made with the controls. Intact cell counts throughout the incubation period showed that all of the studied microorganisms were able to grow in fresh, open culture urine, in competition with native microbes by continually measuring higher ICCs in inoculated tests. Growth rates of the inoculated microorganisms were negligible after 2 days of incubation, indicating that the stable phase of growth had been reached in inoculated tests and controls. Growth rates in sterile sludge dewatering liquors of the trialled microbes were between 0.02 and 0.14 L/h^26,29^ and reached 0.18 L/h in urine inoculations, supporting the hypothesis that fresh urine is a good substrate for the growth of the selected microorganisms. This was further supported by HNA/LNA measurements, which showed inoculated tests had a much higher proportion of HNA to LNA during the first 4 days of incubation (Figure 2). High nucleic acid is indicative of actively metabolizing and replicating cells producing more DNA and RNA,^41^ which strongly suggests the inoculated microorganisms were responsible for the majority of measured growth, in tests during the first 4 days of incubation. Past studies have demonstrated and identified *B. pumilus, M. xanthus, B. antiquum*, and *H. salinarum* as urease producing microbes.^29^ As such, the results support the hypothesis stipulated at the start of this experiment and that fresh urine is a successful growth media for the studied microorganisms as it provides a suitable carbon source in the form of urea and is relatively sterile compared to municipal wastewater.

In the inoculated tests, there were clear differences in sCOD, PO_4_–P, Mg^2+^, and NH_4_–N concentrations over the 10-day incubation period compared to controls. This was the most obvious by day 4 of incubation (Figure 3 and Figure 4) where PO_4_–P removal exceeded controls by up to 49% and NH_4_–N production was 4.6 times greater in *B. antiquum* and at least 2 times greater in all other inoculated batches. The PO_4_–P removal by *B. antiquum* and *B. pumilus* fell within the range of 63% to 76% PO_4_–P removal measured within B4.1 growth media.^28,29^ Additionally, Mg^2+^ removal was similar to those seen after 5 days of incubation in B4.1 synthetic media, where removal was up to 96%.^29^ This supports the hypothesis that fresh urine provides the necessary nutrients and conditions for the studied microorganisms to achieve high nutrient removal rates and PO_4_–P recovery.

The observed increase in NH_4_–N in inoculated tests is indicative of urea hydrolysis. *Idiomarina loihiensis* was the only microorganism not to produce urease^29^ and measured the least ammonia production and ureolysis. All other microorganisms tested (except *Idiomarina loihiensis)* have been shown to produce urease^29^ which accelerated the ureolysis compared to controls in this study. Most notably, *B. antiquum* hydrolyzed at least 34% of the urea present after 10 days. This rate of ureolysis is only matched or exceeded abiotically when adding reagents and additional enzymes and heating to >50°C which can bring complete ureolysis down to several hours.^14,24^ The first step of urine ureolysis is needed for chemical struvite recovery and other approaches in the treatment of urine (Table 1). The increased NH_4_–N is beneficial for struvite reaction kinetics as it raises the molar ratio of N in M:N:P needed for struvite crystal growth,^42^ and the resulting increase in the pH due to urea hydrolysis means that struvite precipitates would remain stable without the need to make pH adjustments.^1,42^ The ability of 4 of the microbes to produce urease means that this step can be completed without chemical addition^29^ and as a result cause biologically induced mineralization of struvite for *B. pumilus, H. salinarum*, and *M. xanthus*. *Brevibacterium antiquum* is able to induce struvite mineralization (BIM) through urea hydrolysis by producing urease and exhibited the greatest ureolysis of all microbes measuring a 6.7-fold increase in 4 days; additionally, it is able to concentrate ions intracellularly to control the bio-mineralization of struvite (BCM).^28^ Additionally, this NH_4_–N-rich supernatant offers opportunities for secondary treatment and targeted ammonia recovery through means of stripping or ion exchange.^43,44^

Magnesium addition to *B. antiquum* tests showed up to 97% PO_4_–P could be removed, and in such tests, it was also observed improved sCOD removal when the ratio of Mg:P was 1:1. To achieve maximum chemical struvite recovery, the ideal Mg:P ratio is 2:1;^1,11,12^ this finding suggests that half the Mg^2+^ dosing would be required to completely remove PO_4_–P using BCM struvite recovery with *B. antiquum*. Additionally, no negative impact on the growth of *B. antiquum* was observed. Another benefit is that the purity Mg^2+^, if added as a supplement, may be of lower criticality, as the biological route for nutrient recovery through bio-mineral formation is likely to be less impacted by competing ions and lower saturation indices, when compared with traditional chemical recovery.^1,11−14^

Modelling and mass balances allowed the calculation of saturation indices of minerals likely to precipitate in urine; and it was demonstrated that abiotic struvite formation was not favorable in any of the urine batches, whereas calcium phosphates (principally hydroxyapatite) were likely to precipitate based on the positive saturation indices. This indicates that without the biological mechanisms behind bio-mineral formation, struvite precipitation would not have been thermodynamically possible. The yields of the recovered precipitates > 10 μm in inoculated tests were greater than controls by up to 37-fold (*B. pumilus*). Compared to recovery yields from B4.1 growth media, yields were 66% lower on average 1500 mg struvite per L B4.1 media^28,29^ compared to an average yield of 456 mg struvite/L urine. *Bacillus pumilus* struvite yield was 33% lower than that of B4.1 media. The limiting Mg^2+^ cations and production of abiotic calcium phosphate are likely the cause for reduced yields in struvite. The recovery of precipitates significantly surpassed the quantities retrieved from SDL by up to 6 times.^29,31^ This finding is promising for application to urine-only treatment and nutrient recovery as it shows recovery can become more efficient by source separating urine, as seen in life cycle assessments of other urine-only treatment techniques.^5^ Precipitate analysis contradicts the calculated saturation indices for inoculated tests, which indicated that struvite precipitation was unfavorable throughout the incubation period. Mineralogical analysis showed that bio-struvite was clearly recovered from all inoculated tests and that the proportion of bio-struvite to Ca-minerals was greater than stoichiometric mass balances suggested. This finding provides evidence that the inoculated microorganisms were able to overcome thermodynamic constraints to produce bio-struvite through their BCM and BIM mechanisms.^29^ Furthermore, greater proportions of bio-struvite to Ca-minerals recovered from inoculated tests indicate that once nucleated due to BCM/BIM, crystal aggregation and growth of bio-struvite will continue. The greatest yield of bio-struvite was from bottles inoculated with *B. antiquum* and *B. pumilus*; interestingly, these microorganisms use BCM and BIM mechanisms respectively to precipitate bio-struvite,^28^ suggesting the mechanism of bio-mineral formation did not influence the quantity of bio-struvite recovered from urine. Whether BIM or BCM is better for biological phosphorus removal and recovery as struvite remains to be investigated in more complex wastes such as sludge dewatering liquors, where the interaction of suspended solids could inhibit the nucleation of BIM of struvite. When Mg^2+^ was added to the bottles inoculated with *B. antiquum*, precipitate yields increased 3-fold in the same incubation period which is promising for improving the efficiency of PO_4_–P recovery from urine and also for pilot and industrial scale studies.

These results show promise for the application of the bio-mineral formation technology to source-separated urine to recover bio-struvite, in a one-step process compared to multistage treatment methods such as complete urea hydrolysis or stabilization before chemical or physical treatment can occur (Table 1). Other main advantages of the bio-mineral formation technology include natural pH increase/stabilization, sCOD removal, and bio-struvite being selectively produced, with minor proportions of calcium based precipitates. Dosing of Mg^2+^ is not necessary to obtain bio-struvite production, but to obtain full PO_4_–P removal, Mg^2+^ should be dosed with the source of Mg^2+^ and the P:Mg ratio deserving more research. In this study, 96% PO_4_–P removal was obtained, and this could be further optimized. In chemical derived processes focused on struvite precipitation, the addition of Mg^2+^ is needed to initiate the crystallization process and produce sizable crystals with PO_4_–P recovery between 60–93%.^8^ The bio-struvite purity was not analyzed in this study, but recent research conducted with sludge dewatering liquors demonstrated that pathogens and micropollutants were not detected.^31^ It is challenging to compare the microorganisms tested side by side, due to the variable characteristics of the urine at the start, but B. *antiquum*, *B. pumilis*m. and *M. xanthus* presented interesting features and results overall and should be further investigated.

Developing these results to larger scale experiments can lead to sustainable, environmentally diligent nutrient recovery from wastewater and urine to secure recoverable fertilizers, improve food security, and develop biobased circular economy. For larger scale applications, the bio-struvite formation can take place in a process fed by fresh urine. If the urine collection or transport systems are in place, these can promote urea hydrolysis, and this should be favorable; but the pH’s should be maintained below 9 before to avoid precipitation of other salts and promote the formation of bio-struvite.^34^

## Data Availability

Data underlying this study can be accessed through the Cranfield University repository at https://doi.org/10.17862/cranfield.rd.25672368.

## Supporting Information Available

Table with pH measurements of inoculated bottles and their respective controls over time; ICC data from flow cytometry analysis for *B. antiquum*, *B. pumilus*, *H. salinarum*, *I. loihiensis*, and *M. xanthus* and their respective control; variation of sCOD, total nitrogen, ammonia, and orthophosphate over the incubation period; and weight of precipitate recovered over 10 days of incubation (PDF)
