# Supplementary material for: Nutrient Removal and Recovery from Urine Using Bio-Mineral Formation Processes
Source: ACS Sustain Resour Manag. 2024 Sep 13;1(9):1906–18. doi: 10.1021/acssusresmgt.4c00025 (PMC11440639; doi:10.1021/acssusresmgt.4c00025)
Supplement: Supplementary file 1 — rm4c00025_si_001.pdf [file rm4c00025_si_001.pdf]

Nutrient removal and recovery from urine using bio-mineral formation processes

Robert E. Colston[a], Ajay Nair[b], Peter Vale[c], Francis Hassard[a], Tom Stephenson and Ana Soares[a]

[a]Cranfield Water Science Institute, Cranfield University, College Road, Cranfield, MK43 0AL, UK

[b]Microvi Biotech, 26229 Eden Landing Rd, Hayward, CA 94545, United States

[c]Severn Trent Plc. Severn Trent Centre, 2 St John's Street, Coventry, CV1 2LZ

Supplementary information

Table Si-1. pH measurements of inoculated bottles and their respective controls

Time (days)	0	1	2	4	7	10
B. antiquum	6.4 ±0.0	7.3 ±0.0	8.8 ±0.0	9.5 ±0.0	9.4 ±0.1	9.5 ±0.1
**UB1**	6.4 ±0.0	7.0 ±0.0	7.3 ±0.0	7.4 ±0.2	8.3 ±0.3	8.8 ±0.1
B. pumilus	6.1 ±0.0	6.3 ±0.0	6.8 ±0.0	7.6 ±0.1	8.5 ±0.1	8.8 ±0.1
**UB2**	6.1 ±0.0	6.3 ±0.0	6.6 ±0.0	8.0 ±0.1	8.6 ±0.1	8.9 ±0.1
H. salinarum	5.8 ±0.1	6.1 ±0.0	6.9 ±0.1	7.3 ±0.0	7.5 ±0.1	7.1 ±0.0
**UB3**	5.8 ±0.0	5.8 ±0.1	5.9 ±0.1	6.5 ±0.2	6.9 ±0.1	7.4 ±0.2
I. loihiensis	5.9 ±0.0	6.7 ±0.3	7.5 ±0.0	7.9 ±0.0	8.7 ±0.2	9 ±0.0
**UB4**	5.9 ±0.0	6.3 ±0.0	6.4 ±0.1	6.7 ±0.1	7.2 ±0.2	7.3 ±0.2
M. xanthus	6.1 ±0.0	6.8 ±0.0	7.0 ±0.0	7.4 ±0.1	7.9 ±0.0	8.3 ±0.2
**UB5**	6.1 ±0.0	6.4 ±0.0	6.5 ±0.0	6.5 ±0.1	7.1 ±0.1	7.3 ±0.1

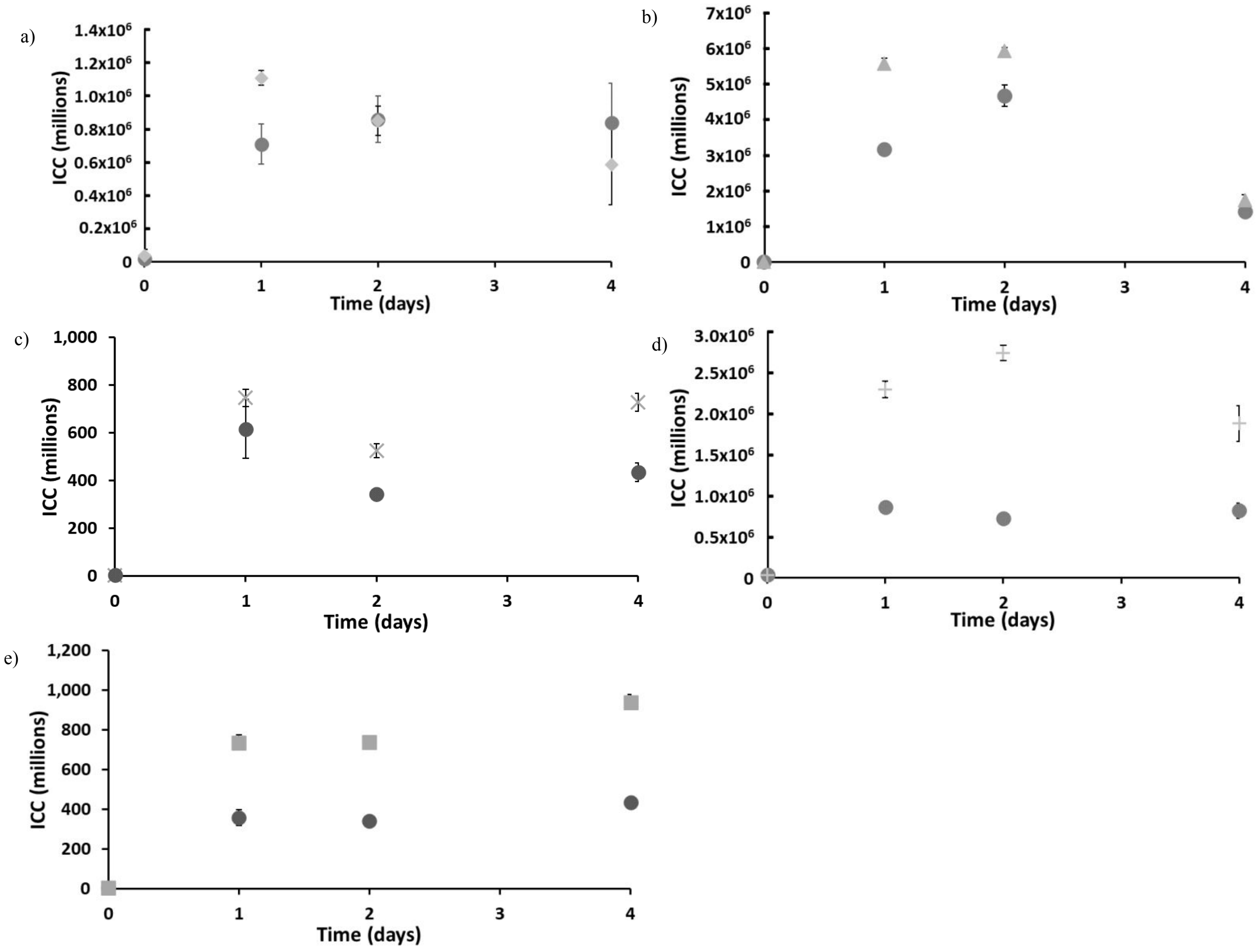

Figure Si-1. Inoculated bacteria and control ICC data from flow cytometry analysis for a) *B. antiquum,* b) *B. pumilus,* c) *H. salinarum,* d) *I. loihiensis* and e) *M. xanthus* and their respective control (●).

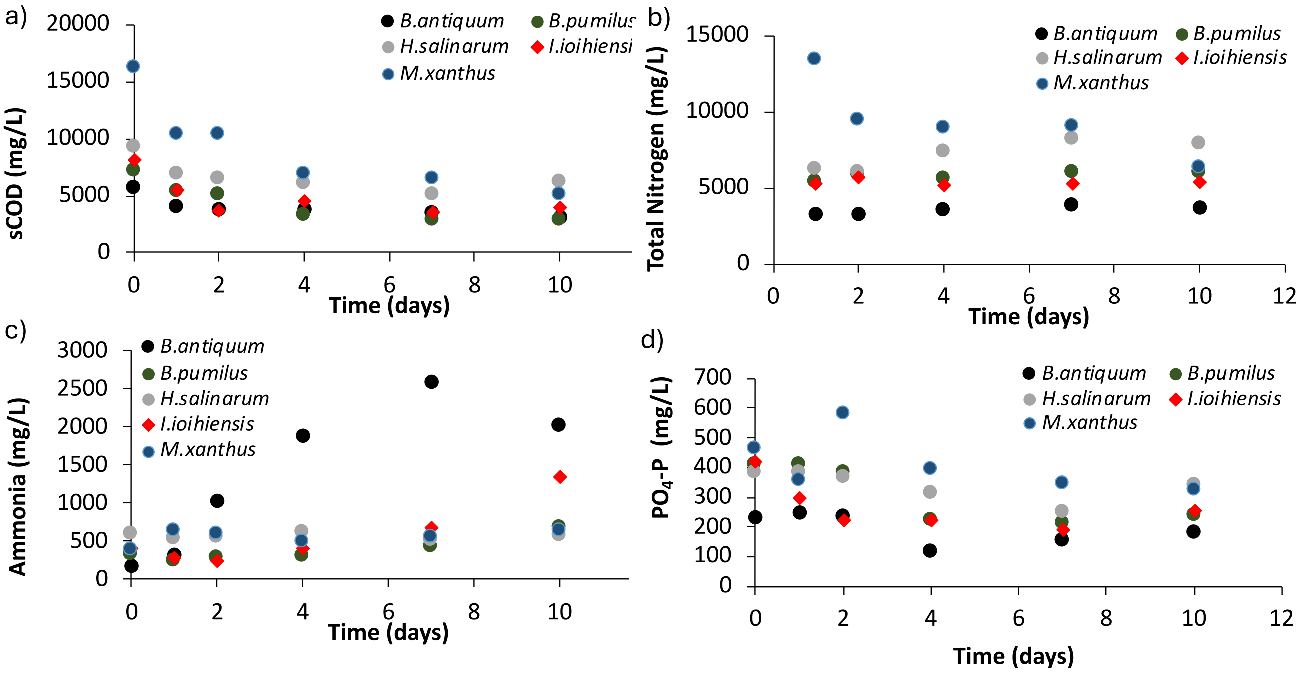

Figure Si-2. Variation of a) sCOD, b) total nitrogen, c) ammonia and d) orthophosphate over the 10 days incubation with selected microorganisms in fresh urine.

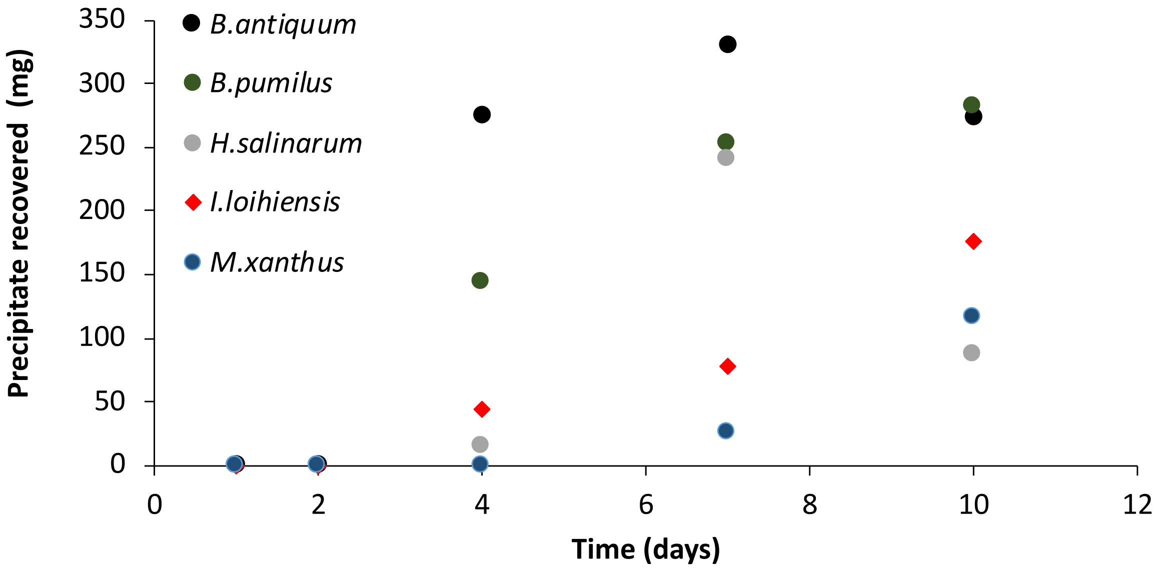

Figure Si-3. Weight of precipitate recovered over the 10 days incubation with selected microorganisms in fresh urine.
